# Clinical Correlation of the Folts Model of Ischemia in a Middle-Aged Woman With Poorly Controlled Type 2 Diabetes

**DOI:** 10.14740/jmc5326

**Published:** 2026-07-01

**Authors:** Varshitha Tumkur Panduranga, Serena Joseph, Joseph Jayaraj, Amin Agah, Jonathan D. Marmur, Louis Salciccioli

**Affiliations:** a Department of Medicine, SUNY Downstate Medical Center, Brooklyn, NY, USA; b Department of Cardiovascular Medicine, SUNY Downstate Medical Center, Brooklyn, NY, USA

**Keywords:** Coronary angiography, Folts model of ischemia, Diabetes mellitus, PCI

## Abstract

Folts model of ischemia describes cyclic flow reduction (CFR) resulting from interaction of severe vessel stenosis and super-imposed episodic platelet-rich thrombus formation. Episodic platelet aggregation at the stenotic site produces partial or near-total obstruction, causing transient ischemia that resolves spontaneously without persistent vessel occlusion. Here we present a case of 50-year-old woman with uncontrolled type 2 diabetes with severe chest pain following weeks of brief, self-resolving episodes at rest and with exertion. Despite serially negative high-sensitivity troponins and nonspecific T-wave changes on 12-lead electrocardiogram, her high-risk features prompted angiography, which revealed a critical 95% mid-left anterior descending artery stenosis. The pattern of recurrent, transient ischemic symptoms without biomarker elevation closely reflected Folts’s model of CRF, in which severe fixed stenosis promotes episodic platelet-mediated obstruction. Intravascular ultrasound-guided percutaneous coronary intervention with drug-eluting stent placement restored normal flow, and her symptoms resolved. This case illustrates how clinical unstable angina can mirror the physiologic principles described in Folts’s model and underscores the need for early invasive evaluation even in the absence of infarction.

## Introduction

The pathophysiology of recurrent ischemia in the absence of infarction was elucidated by the experimental work of Folts and colleagues, who demonstrated that critical coronary narrowing promotes episodic platelet-rich thrombus formation, transiently reducing coronary blood flow [1, 2]. In this model, the platelet plug repeatedly forms over the stenotic lesion and then fragments spontaneously, restoring flow. These recurring cycles of partial obstruction and reperfusion produce short-lived ischemia without complete vessel occlusion, closely resembling the clinical pattern of unstable angina [3, 4]. These findings established platelet activation and dynamic thrombus formation as central drivers of ischemia prior to overt infarction [1, 3]. This mechanism provides a physiologic basis for unstable angina in the absence of myocardial necrosis.

Here we present a case of a 50-year-old woman with poorly controlled type 2 diabetes mellitus whose clinical course closely mirrors these experimental principles. She experienced weeks of recurrent, self-resolving chest pain occurring at rest and with exertion—a pattern consistent with episodic platelet-mediated flow reductions superimposed on severe fixed coronary stenosis. Despite serial high-sensitivity troponins remaining negative and electrocardiographic changes remaining nonspecific, coronary angiography revealed a critical 95% mid-left anterior descending artery (LAD) stenosis. Intravascular ultrasound (IVUS)-guided percutaneous coronary intervention (PCI) with drug-eluting stent placement restored normal coronary flow and resulted in complete symptom resolution. Importantly, this case also illustrates how poorly controlled diabetes mellitus—through its well-established pro-thrombotic and platelet-hyperreactive effects—may further amplify the frequency and severity of cyclic flow reductions (CFRs) at a critical stenosis, thereby lowering the threshold for clinically manifest unstable angina.

## Case Report

A 50-year-old woman with a history of uncontrolled type 2 diabetes mellitus (T2DM) presented to the emergency department (ED) with severe retrosternal chest pain. On presentation, the pain had acutely begun at home while she was at rest, and was described as a pressure-like sensation, radiating to the back and right side of the chest, lasting approximately 40 min, and associated with dyspnea. Over the preceding 2 weeks, she had experienced similar but less intense episodes of chest pain intermittently. These prior episodes occurred both at rest and during exertion, were self-limited, and had not been previously evaluated. She took a single dose of aspirin at home on the advice of her sister, which partially improved her pain. She also self-administered two doses of her partner’s metoprolol, resulting in mild further improvement.

Her past medical history was notable for T2DM diagnosed in 2020. She had initially been treated with metformin and a glucagon-like peptide-1 (GLP-1) receptor agonist for approximately 1 year but had self-discontinued both medications and had been off medications for almost a year. She had a remote history of gestational diabetes mellitus in 2000. Her glycated hemoglobin (HbA1c) was 12%, consistent with poorly controlled diabetes mellitus.

There was no documented history of coronary artery disease, prior myocardial infarction, or cardiac procedures. She denied smoking, use of alcohol or other illicit drugs.

On arrival to the ED, she was afebrile with a temperature of 36.8 °C. Blood pressure was 179/95 mm Hg, heart rate 103 beats per minute, respiratory rate 19 breaths per minute, and oxygen saturation 97% on room air. Her physical examination was notable for tachycardia and normal S1, S2. However, no murmur, gallops, rubs, raised jugular venous pressure or pedal edema was noted.

Initial laboratory evaluation showed a markedly elevated random blood glucose of 435 mg/dL. Serial high-sensitivity troponin measurements, obtained at presentation and at 3- and 6-h intervals, remained negative throughout. N-terminal pro-B-type natriuretic peptide (proBNP) was 125 pg/mL. Other routine blood tests, including complete blood count, renal function, electrolytes, and liver function tests, were within normal limits. Twelve-lead electrocardiogram (ECG) demonstrated sinus rhythm with non-specific T-wave flattening in the lateral leads (I, aVL, V4–V6), without ST-segment elevation or depression. Chest radiography did not reveal any acute cardiopulmonary pathology. Despite the absence of diagnostic ST segment changes or biomarker elevation, the patient’s recurrent chest pain at rest and with exertion in the setting of a higher cardiovascular risk profile with uncontrolled diabetes and symptom progression, was consistent with a clinical diagnosis of unstable angina.

Given the recurrent chest pain, cardiovascular risk profile, and ECG changes, she was treated as having unstable angina. In the ED and early during admission, she received aspirin, nitroglycerin, and insulin for hyperglycemia, with improvement in chest discomfort and blood glucose levels.

She was admitted with continuous cardiac monitoring for unstable angina workup. Transthoracic echocardiography (TTE) performed during hospitalization showed a left ventricular ejection fraction of 62% with normal left ventricular wall thickness, no regional wall motion abnormalities, and no significant valvular disease. Given her recurrent symptoms, high-risk clinical features including poorly controlled diabetes, and concern for unstable angina despite negative biomarkers, an early invasive strategy with coronary angiography was pursued in accordance with guideline-directed management.

Prior to the procedure, she was initiated on dual antiplatelet therapy with aspirin and ticagrelor, intravenous unfractionated heparin infusion, and high-intensity statin therapy with rosuvastatin.

**Figure 1:**
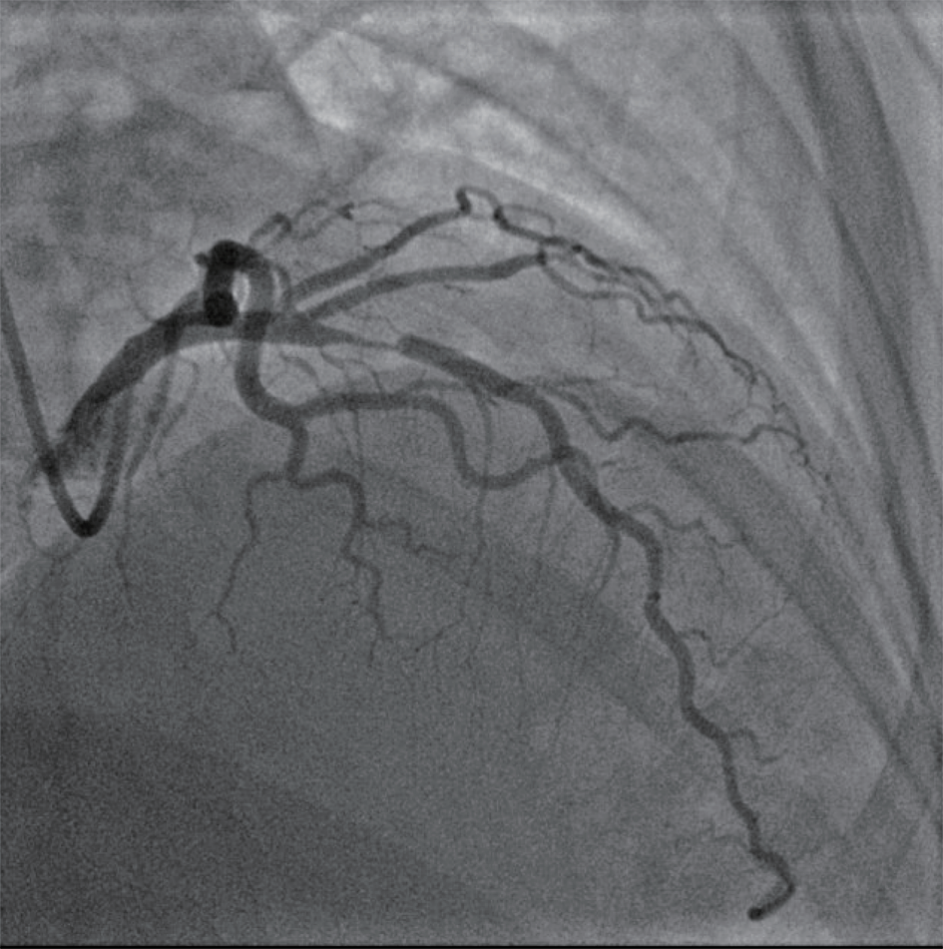
Coronary angiography demonstrating a severe, smooth 95% stenosis of the mid segment of the left anterior descending coronary artery (LAD). The lesion has a concentric, non-calcified appearance without angiographic evidence of dissection, intraluminal thrombus, or ulceration. Preserved distal vessel filling is noted, consistent with high-grade fixed stenosis without complete occlusion. This degree of luminal compromise exceeded the critical threshold for flow-limiting obstruction, creating the substrate for episodic platelet-mediated cyclic flow reductions as described in the Folts model.

**Figure 2:**
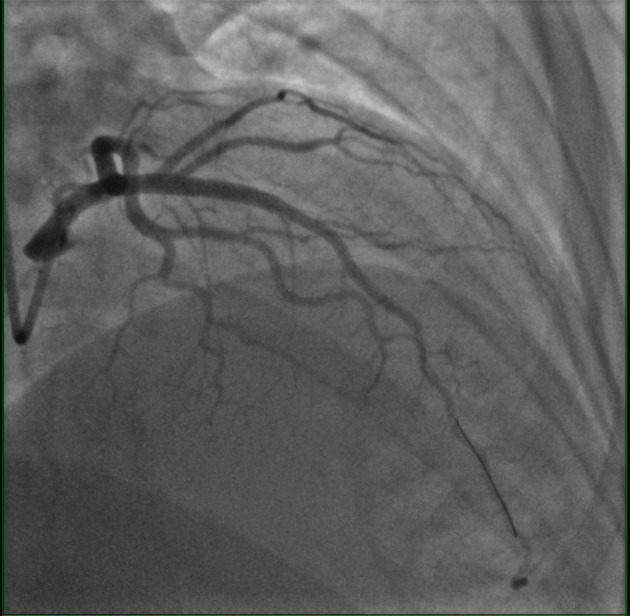
Post-intervention coronary angiography following intravascular ultrasound (IVUS)-guided percutaneous coronary intervention (PCI) with placement of a single drug-eluting stent in the mid left anterior descending coronary artery (LAD). The stent demonstrates complete expansion without geographic miss, residual stenosis, or edge dissection. TIMI-3 anterograde flow is restored throughout the mid and distal LAD and its diagonal branches, with no evidence of distal embolization or no-reflow phenomenon. The post-dilation result, achieved with a 4.0 × 15 mm non-compliant balloon at 14 atmospheres, confirms adequate luminal gain and stent apposition.

Coronary angiography revealed one-vessel coronary artery disease with a 95% stenosis of the mid segment of the LAD (Fig. 1). The lesion appeared angiographically as severe, smooth stenosis without calcification. The patient underwent IVUS-guided PCI with placement of a single drug-eluting stent in the mid LAD. Post-dilation was performed using a 4.0 × 15 mm non-compliant balloon inflated to 14 atmospheres. Post-intervention angiography demonstrated good stent expansion with restoration of normal distal coronary flow and no significant residual stenosis (Fig. 2). No immediate procedural complications were noted.

In the post-procedure period, she remained hemodynamically stable without recurrent chest pain. Management focused on aggressive control of her uncontrolled diabetes mellitus. Given longstanding non-adherence to diabetic medications, and the markedly elevated blood glucose at presentation, her insulin regimen was escalated, with a focus on establishing a sustainable outpatient regimen and reinforcing diabetes education. She was discharged with dual antiplatelet therapy (aspirin and ticagrelor), high-intensity statin therapy, and an intensified insulin regimen, along with close outpatient cardiology and primary care follow-up.

## Discussion

This case describes a 50-year-old woman with longstanding, poorly controlled T2DM who presented with a culminating episode of severe retrosternal chest pain superimposed on a 2-week history of recurrent, self-resolving ischemic episodes at rest and with exertion. The episodic, biomarker-negative nature of her symptoms—with transient relief from aspirin and beta-blockade prior to arrival—raised immediate clinical concern for high-risk unstable angina with an underlying dynamic obstructive mechanism. Serial high-sensitivity troponin measurements remained negative throughout her hospitalization, and electrocardiographic changes were nonspecific, illustrating the diagnostic challenge inherent to biomarker-negative ischemia. TTE demonstrated preserved left ventricular systolic function without regional wall motion abnormalities, further underscoring that transient ischemia had not yet progressed to infarction. Coronary angiography revealed a critical 95% mid-LAD stenosis with a smooth, concentric, non-calcified morphology, and IVUS-guided PCI with drug-eluting stent placement achieved complete stent expansion, restored TIMI-3 anterograde flow, and eliminated her symptoms. Her markedly elevated blood glucose of 435 mg/dL at presentation reflected severe, prolonged glycemic dysregulation resulting from self-discontinuation of all antidiabetic medications nearly 1 year prior. The convergence of critical fixed coronary stenosis and a profoundly pro-thrombotic diabetic milieu in this patient represents a compelling real-world illustration of the Folts model of cyclic coronary ischemia, wherein recurrent platelet-mediated thrombus formation over a flow-limiting lesion produces episodic ischemia without persistent vessel occlusion or myocardial necrosis.

James Douglas Folts was a cardiovascular physiologist who developed what is now known as the Folts model of ischemia during the 1970s and 1980s. Folts and his colleagues mechanically produced a critical coronary artery stenosis of greater than 70–80% and created endothelial injury downstream of the stenosis in anesthetized dogs, establishing a substrate conducive to platelet aggregation [1, 2]. Using continuous Doppler flow measurements, they observed repeated episodes of progressive coronary blood flow reduction caused by platelet-rich thrombus formation at the injured site, followed by spontaneous embolization of the platelet plug with transient restoration of flow [1]. This pattern of CFRs recurred every few minutes and closely simulated the fluctuating coronary perfusion characteristic of unstable angina and non-ST elevation ischemia [1, 3].

There are three important inferences from the Folts model. First, platelets play a central role in acute ischemia, as CFRs were markedly diminished when platelet activation was suppressed with agents such as aspirin or ibuprofen, confirming that platelet aggregation—rather than fixed stenosis—was the immediate trigger for ischemia [5, 6]. Second, coronary thrombosis is a dynamic process in which platelet-rich thrombi form and embolize repeatedly, producing the oscillating perfusion patterns and fluctuating symptoms characteristic of unstable angina [7, 8]. Third, antiplatelet therapies interrupt this cycle by reducing platelet aggregation and attenuating cyclic flow variations, a finding that established the Folts model as a foundational experimental platform for evaluating antiplatelet agents before human clinical trials [5, 6, 9].

The patient’s 95% mid-LAD stenosis exceeded the threshold at which a fixed lesion becomes capable of producing flow-limiting obstruction, a concept supported by early studies linking severe stenosis with impaired hyperemic flow [10]. Crucially, it is the dynamic, recurrent thrombotic process superimposed on this fixed stenosis—rather than the anatomic obstruction alone—that accounts for the episodic and reversible nature of ischemic symptoms. Her clinical pattern—brief, recurrent episodes of chest discomfort occurring both at rest and with exertion, followed by spontaneous relief—closely resembled the cyclic ischemia demonstrated in Folts’s experimental work. In this model, platelet activation over a critical stenosis drives episodic thrombus formation, causing transient flow reduction that reverses spontaneously upon embolization of the plug [1, 2, 6]. Because these interruptions are short-lived, they typically do not produce sustained myocardial necrosis, and biomarkers may remain normal, consistent with the patient’s repeatedly negative high-sensitivity troponin values [3, 8]. This distinguishes unstable angina from non-ST-elevation myocardial infarction (NSTEMI), in which sustained ischemia leads to myocyte necrosis and detectable troponin elevation; in the Folts model, the brevity of each ischemic episode allows reperfusion before irreversible myocardial damage occurs. Her partial symptom improvement after a single aspirin dose at home directly illustrates the underlying platelet inhibition mechanism: by blocking thromboxane A2-mediated platelet activation, aspirin attenuated the recurrent platelet plug formation responsible for episodic flow reduction, consistent with the antiplatelet effects observed in the Folts model [1, 5–7]. Subsequent stabilization following aspirin and nitroglycerin administration in the ED further reinforced this mechanism. The overall clinical picture strongly parallels the mechanisms described by Folts and colleagues and aligns with the recognized pathophysiology of early unstable angina [4, 9, 11]. While the Folts model has been extensively described in experimental settings, its direct clinical correlation is infrequently illustrated in contemporary practice, particularly in the era of high-sensitivity troponin assays. This case highlights how classic mechanisms of cyclic flow reduction continue to manifest in real-world patients and emphasizes the importance of recognizing unstable angina even in the absence of biomarker elevation.

Collectively, these abnormalities create a pro-thrombotic state that lowers the threshold for CFRs at a critical stenosis. These factors likely lowered the threshold for cyclic thrombus formation in this patient, facilitating recurrent platelet-rich thrombus formation over the severe LAD lesion and thereby increasing susceptibility to CFRs and the resultant unstable angina [12–14].

In this context, the diagnosis of CFR is inferred from the characteristic pattern of recurrent, self-limited ischemic symptoms prior to intervention and their sustained resolution following revascularization.

### Conclusion

It emphasizes that recurrent symptoms, not biomarkers, should guide timely diagnostic and therapeutic decisions. Early invasive evaluation should be guided by high-risk clinical features rather than biomarker elevation alone.

## Data Availability

Any inquiries regarding supporting data availability of this study should be directed to the corresponding author.
